# Role of the Macrophage Migration Inhibitory Factor in the Pathophysiology of Pre-Eclampsia

**DOI:** 10.3390/ijms22041823

**Published:** 2021-02-12

**Authors:** Tullia Todros, Luana Paulesu, Simona Cardaropoli, Alessandro Rolfo, Bianca Masturzo, Leonardo Ermini, Roberta Romagnoli, Francesca Ietta

**Affiliations:** 1 Department of Surgical Sciences, University of Turin, Via Ventimiglia 3, 10126 Turin, Italy; tullia.todros@unito.it (T.T.); alessandro.rolfo@unito.it (A.R.); 2 Department of Life Sciences, University of Siena, 53100 Siena, Italy; leonardo.ermini@unisi.it (L.E.); roberta.romagnoli@unisi.it (R.R.); francesca.ietta@unisi.it (F.I.); 3 Department of Public Health and Pediatrics, University of Turin, 10126 Turin, Italy; simona.cardaropoli@unito.it; 4 Città della Salute e della Scienza, 10126 Turin, Italy; bmasturzo@cittadellasalute.to.it

**Keywords:** human pregnancy, inflammatory response, cytokines, placenta

## Abstract

Proinflammatory cytokines are produced in pregnancy in response to the invading pathogens and/or nonmicrobial causes such as damage-associated molecules and embryonic semi-allogenic antigens. While inflammation is essential for a successful pregnancy, an excessive inflammatory response is implicated in several pathologies including pre-eclampsia (PE). This review focuses on the proinflammatory cytokine macrophage migration inhibitory factor (MIF), a critical regulator of the innate immune response and a major player of processes allowing normal placental development. PE is a severe pregnancy-related syndrome characterized by exaggerated inflammatory response and generalized endothelial damage. In some cases, usually of early onset, it originates from a maldevelopment of the placenta, and is associated with intrauterine growth restriction (IUGR) (placental PE). In other cases, usually of late onset, pre-pregnancy maternal diseases represent risk factors for the development of the disease (maternal PE). Available data suggest that low MIF production in early pregnancy could contribute to the abnormal placentation. The resulting placental hypoxia in later pregnancy could produce high release of MIF in maternal serum typical of placental PE. More studies are needed to understand the role of MIF, if any, in maternal PE.

## 1. Introduction

Pre-eclampsia (PE) is a syndrome affecting about 5% of all pregnancies [1]. It is a severe complication of human pregnancy, with significant risk of mortality and short- and long-term morbidity for both mother and fetus [2,3]. It is diagnosed by the presence of de novo hypertension after 20 weeks of gestational age accompanied by proteinuria and/or evidence of maternal acute kidney injury, liver dysfunction, neurological features, hemolysis or thrombocytopenia, or fetal growth restriction [4]. Although it is possible to control blood pressure with antihypertensive drugs and to prevent seizures with magnesium sulphate, at the moment the only definitive treatment of PE is timed delivery, often preterm. Preterm birth represents a risk of death or long-term sequelae for the newborn, and the risk increases as gestational age decreases. This inverse relationship between gestational age and risk is a continuum. However, particular attention to weigh the risk for the mother of continuing pregnancy and the risk for the fetus of immediate delivery must be given in cases where the disease occurs before 34 weeks of gestational age [5]. Prevention of PE is feasible for women with known risk factors and is effective only for some forms of the disease [6]. Its etiology is multifactorial and still not completely understood, while there is nowadays evidence that the symptoms are attributable to an excessive inflammatory response that causes generalized endothelial damage [7].

## 2. Immune Response in Pregnancy

Throughout pregnancy, the maternal reproductive system is provided with an inflammatory response essential for the defense against infections. The mucosa of the female reproductive tract is indeed permanently exposed to an extensive variety of microorganisms coming from the external environment and develops protective defense mechanisms [8,9,10]. This implies that the pregnancy is established in an immunologically active environment capable of protecting the mother and the fetus. In support of this activation, the basal expression of cytokines is higher in the mononuclear cells from the female reproductive tissues than in peripheral blood mononuclear cells [11].

To control the microorganisms antigenic load, cells of the female reproductive mucosa act via sensing through pattern recognition receptors (PRRs), a class of evolutionary conserved receptors of which the toll-like receptors (TLRs) are the most studied [12,13]. All known TLRs (1–10) mRNA and protein are expressed in human placenta [14,15,16]. Interestingly, an intense and polarized TLR2 expression was shown in the cell membrane of the villous cytotrophoblast adjacent to the syncytiotrophoblast layer, especially in the first weeks of pregnancy [16]. This peculiar staining pattern together with an abundance of TLR3, TLR4, and TLR5 suggested a TLR defensive barrier against pathogens during the most vulnerable time of fetal development [16,17].

Besides pathogen-associated molecular patterns (PAMPs), such as bacterial lipopolysaccharides (LPS), TLRs also recognize and sense endogenous molecules expelled from injured/damaged tissues, referred to as damage-associated molecules (DAMPs) [18].

**Figure 1:**
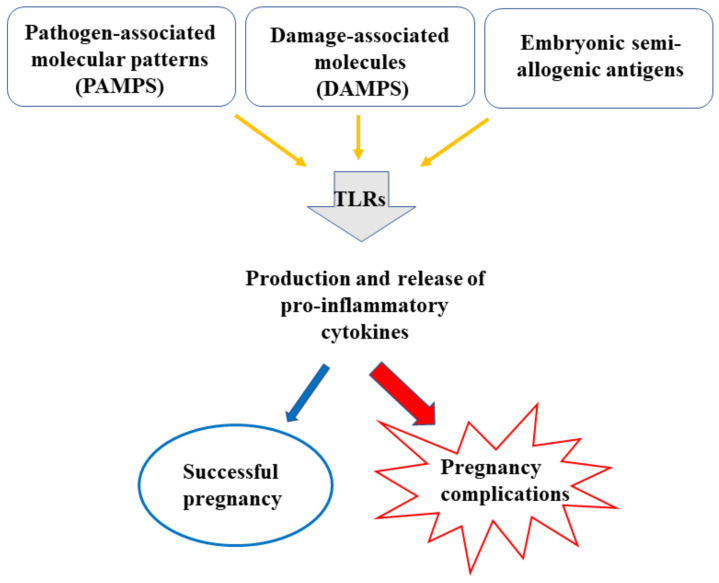
An inflammatory response is essential in pregnancy to protect against infection, repair tissue injury, and promote a tolerogenic milieu for the fetus. An exacerbated inflammatory response can be harmful for pregnancy outcomes causing diseases such as PE.

While responding to microorganisms and/or nonmicrobial causes, a tolerogenic state is, however, needed during pregnancy, to avoid rejection of the semi-allogenic fetus. These apparently conflicting types of response—defense against pathogens and embryo acceptance—are not necessarily separate as they appear to share features of the innate immune response. TLR (1–9) mRNAs are expressed in the decidual macrophages and uterine natural killer (uNK) cells, the major immune cell populations in the maternal uterus [19]. Whatever the triggering signal, activation of TLRs leads to common cascade signals and induces the transcription and the release of proinflammatory cytokines such as TNFα, IL-1, IFNγ, IL-6, and IL-8 [20,21,22] (Figure 1).

Numerous other markers of inflammation are increased in pregnancy: white blood cell count of granulocytes, neutrophils, and monocytes, C reactive protein, erythrocyte sedimentation rate, fibrinogen [23,24,25]. It is, however, important to emphasize that while a controlled and moderate response is essential for successful pregnancy, an exaggerated inflammatory response is implicated in several pathologies including PE [26] (Figure 1). Changes in TLRs have been associated with PE, characterized by higher placental expression of TLR-2, -3, -4, and -9 [27,28].

### 2.1. Normal Pregnancy

If the background picture explains how physiological pregnancy is characterized by an inflammatory response, it is also recognized that the immune condition undergoes different biological phases. A proinflammatory state is present during the earlier and later phase of pregnancy while an anti-inflammatory one characterizes the intermediate period [29,30,31,32]. This concept, developed approximately in the last two decades, is in contrast to the hitherto established idea which viewed pregnancy as a single event with an anti-inflammatory or immune suppression state. This theory postulated that a successful pregnancy is sustained by type 2 T-cell (Th2)-derived cytokines and a shift to Th1 cytokines would lead to early abortion or pregnancy disorders [33,34,35]. It is now currently recognized that the Th2/anti-inflammatory cytokines are important mediators for the maintenance of an already established pregnancy, allowing uterine quiescence and fetal growth and development [30,32]. On the other hand, a predominance of Th1/proinflammatory cytokines is needed in the earlier and later stage events [36]. The complex of immunomodulatory molecules secreted by both embryonic and maternal tissues exerts its action on complementary tissues acting as communication signals between mother and fetus, since very early embryonic development [37]. During this first stage, the blastocyst breaks through the uterine epithelium in order to implant; the trophoblast cells that line the blastocyst—and more specifically the extravillous trophoblast cells—migrate within and around the mother’s blood vessels and replace the endothelium and vascular smooth muscles. As a result, the spiral arteries become unresponsive to endocrine and vasoactive stimuli, thus attaining the physiologic properties that are required to adequately perfuse the placenta [38]. All these activities, involving the contribution of proangiogenic factors, require an inflammatory environment capable of repairing the tissue, producing vascular changes, and creating an appropriate uterine immune response for the acceptance of the semi-allogenic fetus [39]. The maternal decidua is filled with a unique immune cell population of which the NK cells with a higher cytokine profile and poor cytotoxic potential are the most abundant [40,41]. The proinflammatory environment decays approximately at the end of the first trimester, to increase again in approximately the event of labor, contributing to uterine contractions and cervical ripening [42]. The local cytokine profiles at the maternal–fetal interface guarantee fetal growth and development on the one hand and have profound reflexes on the mother’s physiological adaptations to pregnancy on the other hand [43]. In fact, during pregnancy, striking functional changes occur in the maternal organism, involving cardiovascular, hematologic, renal, respiratory, gastrointestinal, endocrine, and metabolic systems [43].

### 2.2. Pre-Eclampsia

In pre-eclampsia, all the markers of inflammation as well as circulating levels of pro-inflammatory cytokines and chemokines are increased compared with physiological pregnancy [7,25,44,45,46,47]. The generalized inflammatory response with high levels of cytokines, such as IL-1β, IL-8, IL-6, TNFα, may occur in the absence of a microbial infection: potential stimuli can arise from cellular stress, trophoblast necrosis or apoptosis, placental hypoxia [48]. However, also an infection already established before pregnancy or developing during pregnancy may be the trigger for the development of PE [44,49,50,51,52,53]. Women affected by autoimmune diseases, such as antiphospholipid syndrome and systemic lupus, are at high risk of developing PE, strongly suggesting that a dysregulation of the immune system plays a crucial role in the etiopathogenesis of the disease. Also, the higher incidence of PE in primigravidas, or those who have limited contact with paternal antigens, indicates the involvement of the immune system. On the other hand, the increased risk of PE in women with diseases directly or indirectly affecting the cardiovascular system underscores that the inability of the maternal organism to cope with the needs of the growing placenta and fetus contributes to PE. It is thus clear that PE is a multifaceted, multifactorial syndrome sharing pathophysiologic mechanisms (systemic inflammation, oxidative stress) with non-pregnancy disorders such as hepatorenal syndrome, cardiorenal syndrome, cardiohepatic syndrome, as it is well outlined in a recent review by Gyselaers [54]. Recently common features (cytokine storm) have been highlighted between the most severe forms of COVID-19 disease and PE [55].

At least two forms of PE are recognized: one of placental origin (placental PE) and one triggered by maternal pre-existing conditions (maternal PE) [56]. Many studies aimed at understanding the etiology of PE show different characteristics of the two populations [53,57,58]. Phenotypically they can be distinguished based on fetal growth: intrauterine growth restriction (IUGR) in placental cases (IUGR-PE) and appropriate for gestational age (AGA) in maternal PE (AGA-PE) [59].

Placental PE more often occurs ≤34 weeks of gestational age [59]. At the basis of placental PE, there is an abnormal interaction between the trophoblast and the decidua mainly occurring during the first trimester of pregnancy. This leads to the pathological development of the placenta: on the maternal side, the vascular bed largely maintains its pre-pregnancy characteristics with the spiral arteries incompletely transformed into dilated unstructured vessels, at variance with normal pregnancy [60]. On the fetal side, the impaired perfusion due to the abnormalities of the maternal vascular bed impacts the growing villi that typically present a reduction in the density of small-stem arteries and in arterial branching [61,62]. The abnormal placenta on the one hand limits the transfer of oxygen and nutrients from the mother to the fetus, resulting in IUGR, and eventually in intrauterine demise [62,63]; on the other hand, it releases molecules that trigger the maternal exaggerated inflammatory response. Trophoblast cells and mesenchymal stromal cells of PE placentae produce and release into the maternal circulation increased amounts of proinflammatory cytokines, chemokines, and antiangiogenic factors compared to placentae from normal pregnancies [45,64], contributing to the endothelial damage and to the clinical picture of the disease.

Maternal PE more often is of late onset (>34 weeks). It is likely to occur in pregnant women with already established conditions that involve an underlying endothelial dysfunction, such as cardiovascular diseases, diabetes, autoimmune diseases, obesity, asymptomatic chronic infections [50,51,65,66]. Therefore, the placenta is not or only secondarily involved, the vascular villous tree has a normal development, and fetal growth is normal.

To differentiate placental and maternal forms of pre-eclampsia may be difficult: it would need a careful study of uterine and umbilical arteries Doppler indices, longitudinal investigation of fetal growth, and eventually of some biomarkers. Therefore, many studies, recognizing different forms of pre-eclampsia, separately report data for cases diagnosed before (early-onset PE) or after (late-onset PE) 34 weeks; this is not the same as “placental” and “maternal”, but it may be a surrogate, since placental PE occurs more often ≤34 weeks and maternal PE more often >34 weeks of gestational age.

## 3. MIF in Normal and Pre-Eclamptic Pregnancy

The cytokine macrophage migration inhibitory factor (MIF) is a pleiotropic inflammatory molecule discovered over half a century ago [67,68]. Originally, MIF was identified as a soluble protein produced by T-lymphocytes capable of inhibiting the random migration of macrophages [67,68]. Today MIF is known as a central regulator of different physiological processes contributing to cell proliferation and differentiation, angiogenic biological activities, and innate immune response [69]. It is normally present in the plasma of healthy subjects at concentrations ranging from 0.1 to 30 ng/mL, produced by a variety of cell types including endocrine, epithelial, endothelial, and immune cells such as monocytes/macrophages, and B and T cells [70,71]. MIF is stored in preformed, cytoplasmic pools and is rapidly released in response to endogenous and/or exogenous stimuli such as microbial products, antigen-specific proliferative signals, and hypoxia [72]. Extracellular MIF acts through interaction with several cell surface molecules, of which CD74, an MHC Class II invariant chain, is the most studied [73,74,75].

Unlike all other known cytokines, MIF has several enzymatic activities, specifically phenylpyruvate tautomerase, L-dopachrome tautomerase, and thiol-protein oxidoreductase activities [76,77]. The major focus of MIF has been on its role in the inflammatory process as a proinflammatory mediator [78,79]. It has been shown that MIF promotes the production and expression of a large panel of proinflammatory mediators including cytokines (TNFα, IL-1β, IFNγ, and IL-6), nitric oxide (NO), and matrix metalloproteases (MMPs) [79,80]. In the context of proinflammatory responses, MIF secretion is induced rather than inhibited by glucocorticoid hormones [81]. Additionally, MIF has the unique ability to reverse the immunosuppressive/anti-inflammatory effects of glucocorticoids [81].

MIF is also an important regulator of innate immune responses and essential for fighting pathogens including Gram-negative bacteria, viruses, and parasites [82,83,84,85,86]. Interestingly, MIF positively regulates the expression of TLR4 in macrophages promoting the recognition of LPS, a cell wall constituent of most Gram-negative bacteria, by the innate immune system [79,87,88]. A very recent report showed that *Mif* gene expression is activated by LPS in *Ciona Robusta*, a marine invertebrate model, suggesting MIF as a universal signaling mediator for the defense against pathogens [89].

MIF response can become exaggerated in many infections, inflammatory, and autoimmune diseases. Accordingly, MIF blood levels are increased in patients with septic shock, systemic lupus erythematosus, and rheumatoid arthritis (see review by Bilsborrow et al. [90]), a feature for which MIF is regarded as a biomarker and a pharmacological target for different diseases [91,92]. Within this context, several classes of MIF inhibitors appear to be of considerable therapeutic benefit in many inflammatory and autoimmune conditions [93,94,95,96,97].

Studies performed in the past two decades showed MIF as one of the immunomodulatory molecules secreted at the maternal–fetal interface [98,99,100]. Although MIF knock-out mice failed to show reduced fertility and produced normal-sized litter [101], extensive evidence supports a high contribution of MIF in pregnancy, particularly during the earlier and later phases, characterized by inflammatory-like events [99,102]. MIF is normally present in tissues and fluids during normal pregnancy including uterus/decidua, placenta, amniotic fluid, and fetal and maternal blood. Maternal MIF serum levels have been shown to be remarkably elevated in pathological conditions such as preterm delivery [103] and pre-eclampsia [44]. High MIF levels might indicate an excessive response in the case of pathological inflammation/infection, which could be harmful to the health of the pregnancy and fetus. The role of MIF in normal and pathological pregnancies is summarized in Table 1 and Table 2.

### 3.1. MIF in Normal Pregnancy

#### 3.1.1. MIF in Early Pregnancy

Among the proinflammatory cytokines implicated in the early phase of pregnancy, MIF is highly involved in placenta establishment and development [98,99,100]. MIF is abundantly present at the maternal–fetal interface secreted both by fetal trophoblast and maternal decidua. MIF mRNA and protein are already present in the uterus during the menstrual cycle mainly in the glandular and surface epithelium, and the stromal and endothelial cells [104]. Quantitative assessment showed a regulated cycle phase-dependent expression pattern with higher levels in the late proliferative/early secretory phase [105,106]. In first trimester trophoblast, MIF is mainly expressed by the cells of the internal proliferative layer of chorionic epithelium and by the extravillous trophoblast [107]. Importantly, trophoblast MIF is upregulated by low oxygen tension, comparable to that occurring in the very early stages of pregnancy because of the absence of maternal blood flow in the intervillous space [108]. Induction of MIF by low oxygen tension was supported by studies in placental tissues from women living at high and moderate altitude, representing a natural in vivo model of chronic hypoxia. The findings showed that the higher the altitude, the higher the concentration of MIF [108].

MIF promotes trophoblast migration and invasion. The abundance of MIF in the earlier phases of gestation has aroused much interest on the role of MIF in the implantation and development of the placenta. The majority of these studies were conducted on HTR-8/SVneo, an in vitro model of cells originated from human first trimester placenta and immortalized by transfection with a cDNA construct that encodes the simian virus 40 large T antigen [120]. These cells are representative of the invasive extravillous trophoblast, specifically of the cells that, detaching from the chorionic villi, migrate to and infiltrate the maternal decidua up to the spiral arteries [120]. A report by Jovanović Krivokuća et al. [109] showed that MIF can act on trophoblasts in an autocrine and paracrine manner. Blocking of endogenous MIF with ISO-1, an inhibitor of the tautomerase activity of MIF, reduced HTR-8/SVneo cell migration and invasion while an opposite effect was obtained by addition of rMIF [109]. Similarly, addition of ISO-1 to decidual stromal cells conditioned media decreased their proinvasive action on trophoblasts [109]. The same research group also studied the potential contribution of trophoblast MIF on the spiral artery remodeling process. They demonstrated that attenuation of endogenous MIF by specific siRNA had a negative effect on the ability of HTR-8/SVneo to differentiate into endothelial-like phenotype [111]. HTR-8/SVneo cells also produce MIF in response to LPS, a cell wall constituent of most Gram-negative bacteria recognized by TLR4 [121]. LPS-treated cells also showed increased levels of MMP-2 and MMP-9 and higher migration activity.

MIF promotes cell survival and suppresses apoptosis. By using the model of chorionic villous explants from first trimester placenta, Ietta et al. showed that MIF is able to protect trophoblasts from excessive apoptosis against hypoxia/reoxygenation injury [110]. Apoptosis is a physiological process in normal placenta development which might become harmful if not properly regulated and lead to pregnancy complications, such as pre-eclampsia and IUGR [122]. Binding of MIF with its receptor CD74 was shown to be an essential mechanism through which MIF suppresses apoptosis [110]. Downregulation of CD74 gene was shown in placenta from women affected by PE [123]. A protective role of MIF was also shown in decidual stromal cells challenged with reactive oxygen species [124]. MIF interferes with the apoptotic fate of these cells by triggering phosphorylation of Mdm2 protein in a PI3K/Akt-dependent manner and changing the nuclear translocation of p53 [124].

MIF in maternal–fetal immunotolerance. NK cells, the major immune cell subpopulation in early pregnancy, produce MIF [99]. MIF also plays an autocrine/paracrine role on these cells by reducing their cytolytic activity, thus contributing to maternal–fetal immunotolerance [106]. Therefore, decidual NK cells are a source and a target of MIF.

Altogether, the data on MIF in early pregnancy are of extreme importance as a deficient placentation may lead to pregnancy disorders such as PE and IUGR [125]. The key role of MIF in establishment of pregnancy and placenta development is supported by the fact that lower MIF secretion in early pregnancy was found to correlate with pregnancy loss [113,114].

#### 3.1.2. MIF from Mid-pregnancy to Term

To our knowledge, only little information is available on MIF from the end of the first trimester to the end of pregnancy. By examining placental tissues at 7–10, 11–12, 14–20 weeks of pregnancy, and at term, Ietta et al. showed that MIF mRNA and protein declined at 11–12 weeks of gestation, then it remained constant until term [108]. By contrast, assessment of MIF concentration in amniotic fluid showed that levels of MIF were increasing from mid-pregnancy (median 20.07 ng/mL) to term (median 62.10 ng/mL) and reached a peak in women at term with labor (median 258.80 ng/mL) [102]. Of note, the changes of MIF in amniotic fluid with advancing gestation were not reflected in maternal serum where levels remained stable from mid-trimester to term and at term with labor. Maternal serum values during pregnancy were not different from values reported in non-pregnant subjects [102]. Other authors reported that MIF maternal serum levels remain unchanged throughout pregnancy, but found values that are higher compared to non-pregnant subjects [118].

#### 3.1.3. MIF in Fetal–Newborn Blood

MIF levels in cord serum at term birth (CS) were found higher than in maternal serum (MS/CS ratio = 0.4), supporting MIF as an inflammatory mediator of labor [102].

In a study at different ages from fetus to adult, Roger et al. showed that the plasma levels of MIF in fetuses at 26–30 weeks were about fivefold higher than in adults and further increased to about 15–20-fold in full-term infants at birth. MIF plasma levels remained high after birth at least until postnatal day 4 and decreased to levels normally found in adults within the first month of life. Based on their data on MIF in regulating neonatal innate immune responses, the authors proposed that high levels of MIF in newborns might play a protective role to reduce susceptibility to infection during the neonatal period [126,127].

### 3.2. MIF in Pre-Eclampsia

Given the role of MIF in the establishment and development of the placenta and its contribution to inflammation/infection response, its study in PE patients may help to explain some of the pathogenetic pathways observed in this disease, which is characterized by exaggerated inflammatory response and/or abnormal placental development. In fact, it has been shown that MIF serum levels in maternal blood and MIF concentration in maternal–fetal tissues are altered in pregnancies complicated by PE at different stages of pregnancy.

#### 3.2.1. MIF in Women Who Later Develop PE

MIF maternal serum levels have been studied in the first half of pregnancy in women who developed PE during the third trimester. Cardaropoli et al. report on MIF levels in the serum of 127 first/early second trimester women, 48 of whom later developed PE. The values were significantly lower in the serum of PE patients than in the ones who had an uneventful pregnancy (4967 ± 1620 pg/mL vs. 7640 ± 5519). However, when two subgroups of PE patients were separately considered, MIF values were significantly lower compared to controls only in 18 patients who developed PE before 34 weeks (3983 ± 1620); in late-onset PE, the values were slightly lower (5557 ± 3642 pg/mL), but not significantly different compared to those in normal pregnancies [119]. In a longitudinal study of 33 pregnancies at high risk of PE, Galbiati et al. measured plasma MIF mRNA at 6–16, 17–23, 24–30, and 31–34 weeks. Nine patients developed PE, three of them ≤34 weeks. They did not find any difference in MIF mRNA at 6–16 and 17–23 weeks between women who developed or did not develop PE, while MIF was significantly higher at 24–30 weeks in women who developed PE. At 31–34 weeks, there was again no difference between the two groups. This could be explained by the fact that the last ones developed late-onset PE. The authors also measured the expression of HIF-1α in maternal plasma and found high levels at 6–16 and 17–23 weeks, thus confirming the important role of oxygen in the pathogenesis of PE [117].

As far as we know, these are the only data on MIF in patients who later develop PE.

There are no data about MIF concentration at the maternal–fetal interface in the first trimester of pregnancy in women who later develop PE. Assuming that low serum levels are paralleled by low MIF concentration at the maternal–fetal interface, one can speculate that it plays a role in the abnormal development of the placenta, since high levels of MIF are required to stimulate trophoblast invasion in early pregnancy. The role of low first trimester MIF maternal serum levels on placental development would also be confirmed by the findings of Yamada et al. They found low MIF serum levels in patients having miscarriage of fetuses with normal chromosomes [113]. First trimester miscarriage of normal embryos/fetuses is due to abnormalities in trophoblast invasion of the decidua, negatively affecting placenta development [125]. Thus, the same placenta abnormalities would lead to abortion when they are extremely severe and more extended, or to PE when they are less severe and less extended.

#### 3.2.2. MIF in Women with Established PE

High levels of MIF in maternal serum of third trimester PE pregnancies were first reported by Todros et al. [44]. The significantly higher levels in PE compared to normal pregnancies (median 12.7 ng/mL vs. 5.3 ng/mL) were due to cases of early-onset PE (17.8 ng/mL); in late-onset PE, the values were comparable to those of controls (6.16 ng/mL vs. 5.3) [44]. The data were confirmed in another study by Cardaropoli et al. where the high values of MIF serum levels in PE pregnancies (5126 *±* 2902 ng/mL vs. 2467 *±* 703 ng/mL) were attributable to cases of PE complicated by IUGR while no significant difference was found between AGA PE and controls [115]. More recently, higher values of MIF maternal serum levels in PE patients were reported by Mahmoud et al. [116]. At variance with all the above data, Hristoskova et al. found that MIF maternal serum levels were increased in normal pregnancy compared to non-pregnant, but they were not further increased in PE patients. However, they subdivided PE pregnancies according to the severity of the disease, which is based on clinical characteristics, but may not reflect different pathophysiological conditions [118]. Therefore, in their population there could be a prevalence of “late-onset PE” where the levels of MIF are not increased.

MIF in placenta. A study by Cardaropoli et al. [115] showed that MIF is expressed in placental tissue from both normal and PE third trimester pregnancies. Differences were observed when IUGR-PE and AGA-PE cases were separately considered. Only in the former was MIF concentration significantly lower compared to placentae from normal pregnancy. Immunoreactivity for MIF was present in the syncytiotrophoblast of all placentae, but only in IUGR-PE placentae was it also present in the intervillous space [115]. When cytokines expression profile expressed by a specific subpopulation of placental cells, placenta-derived mesenchymal stromal cells (PMSCs), was studied in normal and PE placentae (mainly IUGR-PE), a significantly higher release of MIF from PE-PMSCs was shown [45]. However, MIF production by PE-PMSCs does not seem to be sufficient to increase MIF placental content, since these cells show decreased proliferation and increased cellular senescence relative to normal PMSCs [45].

The lower expression of MIF in placental tissue [115] and higher levels in maternal serum [44] could be explained by its increased release in the placenta intervillous blood (IVB) and hence in the maternal blood. High levels of MIF have also been reported in the IVB plasma from women infected with malaria [128]. In a rat model of bladder inflammation, it was shown that MIF concentration is decreased in the endothelium and increased in the bladder lumen [129]. Moreover, in the human it was demonstrated that the influenza A virus infection induces a reduction of MIF in bronchial epithelial cells with an increase in extracellular MIF levels [130]. An alternative explanation could be that the systemic inflammation originated by the hypoxic placenta induces the release of a large amount of MIF by non-reproductive tissues [119].

MIF in fetal membranes. MIF concentration in fetal membranes is significantly higher in AGA-PE cases compared with controls, but not in IUGR-PE [115]. MIF immunostaining was stronger on epithelial cells of amnion side and decidual cells. A similar increase in MIF immunostaining was found in fetal membranes of placentae from pregnancies with malaria infection [131]. This would be further evidence that symptomatic or asymptomatic infections have an etiologic role in the development of PE [44,49,50,51,52,53].

**Figure 2:**
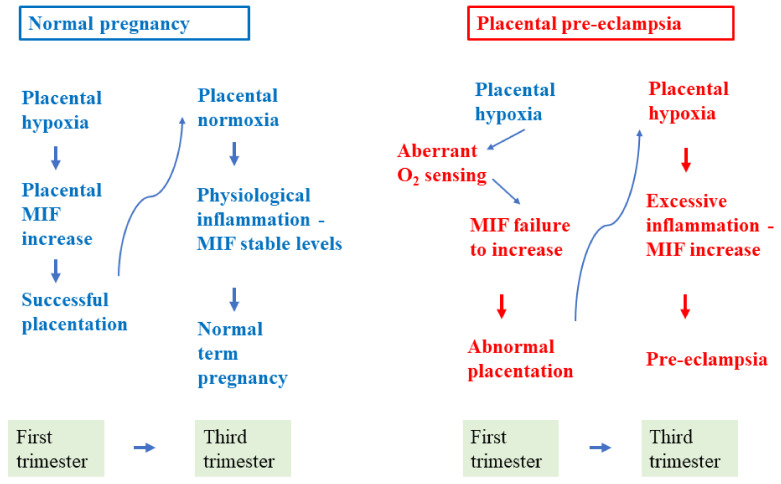
Potential role of MIF in the pathogenesis of placental pre-eclampsia. Low MIF production in early pregnancy would contribute to the abnormal placentation with subsequent placenta hypoxia and increased production of proinflammatory cytokines, placental debris, and oxidative stress leading to pre-eclampsia.

Taken overall, the above data confirm that MIF plays a role in the pathogenesis of PE, but its role is different as different pathogenetic pathways are recognized. The data reported so far led us to speculate that in IUGR-PE and/or early-onset PE, low levels of MIF in early pregnancy contribute to the abnormal placentation, insufficiently stimulating trophoblast invasion; while later in pregnancy, it may induce the general endothelial injury both directly and indirectly by stimulating the production of proinflammatory cytokines (Figure 2). It is less clear which could be its role in AGA-PE and/or late-onset PE as no significant differences compared to controls are reported in early and late pregnancy maternal MIF serum levels or in late pregnancy placental concentration. However, it could play a role in PE of infectious origin, one of the many possible causes underlying this group of PE pregnancies.

## 4. Conclusions

MIF has a role in normal and PE pregnancy. In early pregnancy it contributes to trophoblast invasion and normal development of the placenta. MIF deficiency in the first/early second trimester of pregnancy can lead to miscarriage, or to placental impairment if the pregnancy continues, thus contributing to the pathogenesis of placental PE. It has to be elucidated if the higher levels of third trimester maternal serum MIF are a cause or an effect of the disease. The role of MIF in the pathogenesis of maternal PE is less clear. It might contribute to cases of infectious origin, but its effects can be blunted due to the many other causes involved in the development of this type of PE (metabolic, cardiovascular, autoimmune disorders). Therefore, more targeted studies should be performed to understand its role in maternal PE.

## Figures and Tables

**Table 1:** MIF in human reproduction.

	Findings	References
**Menstrual cycle**	MIF mRNA and protein are expressed in uterine glandular and surface epithelium.	[104]
	Uterine MIF expression is higher in late proliferative and secretory phases.	[105]
**Early pregnancy**	MIF is produced by uNK cells and acts on these same cells by reducing their cytolytic activity.	[106]
	MIF protein is expressed in first trimester placenta mainly in villous and extravillous trophoblast.	[107]
	Trophoblast MIF is induced by hypoxia.	[108]
	MIF promotes trophoblast cell invasion and migration.	[109]
	MIF promotes survival of first trimester human placenta under induced stress conditions.	[110]
	MIF promotes trophoblast differentiation to endovascular phenotype.	[111]
**Mid-pregnancy**	MIF mRNA and protein placenta expression declines at 11–12 weeks and remains stable until term.	[108]
**Term pregnancy**	MIF in amniotic fluid is higher at term than at mid-gestation and higher at term with spontaneous delivery.	[102]
	MIF levels in umbilical cord serum at term birth are higher than in maternal serum.	[102]
	MIF is expressed and secreted by extraembryonic membranes.	[112]

**Table 2:** MIF in pregnancy complications.

	Findings	References
**Miscarriage**	Maternal serum MIF levels in early pregnancy are low in patients having miscarriage.	[113]
	MIF in uterine tissues and maternal blood is low in patients with recurrent pregnancy loss.	[114]
**Pre-term delivery**	Maternal plasma MIF levels at first–second trimester are higher in pregnancies with preterm delivery.	[103]
**Pre-eclampsia**	Maternal serum MIF levels are higher in patients with PE than in normal pregnancy.	[115,116]
	Maternal serum MIF is higher in patients affected by IUGR-PE while not in AGA-PE.	[115]
	MIF mRNA in maternal plasma at 24–30 weeks is higher in patients who later develop PE.	[117]
	MIF maternal serum levels are higher in normal pregnancy compared to non-pregnancy but not further increased in PE patients.	[118]
	Secretion of MIF by placental mesenchymal stromal cells is higher in IUGR-PE than in normal pregnancy.	[45]
	Maternal serum MIF in first–early second trimester is lower in women who later develop PE.	[119]
